# Failure patterns and clinical indications in early-stage follicular lymphoma: a study from the National Cancer Center in China

**DOI:** 10.3389/fphar.2026.1745000

**Published:** 2026-01-14

**Authors:** Chang Xu, Shijie Yang, Yue Liu, Yunpeng Wu, Xin Liu, Qiu-Zi Zhong, Yong Yang, Tao Wu, Si-Ye Chen, Bo Chen, Yong-Wen Song, Hui Fang, Jing Jin, Yue-Ping Liu, Hao Jing, Yuan Tang, Ning Li, Ning-Ning Lu, Wen-Wen Zhang, Shu-Lian Wang, Jingru Zhu, Shu-Nan Qi, Ye-Xiong Li

**Affiliations:** 1 Department of Radiation Oncology, National Cancer Center/National Clinical Research Center for Cancer/Cancer Hospital, Chinese Academy of Medical Sciences and Peking Union Medical College, Beijing, China; 2 Department of Radiation Oncology, Beijing Hospital, National Geriatric Medical Center, Beijing, China; 3 Department of Radiation Oncology, Fujian Medical University Union Hospital, Fuzhou, Fujian, China; 4 Affiliated Hospital of Guizhou Medical University, Guizhou Cancer Hospital, Guiyang, Guizhou, China

**Keywords:** failure, follicular lymphoma, machine learning, radiotherapy, survival outcome

## Abstract

**Background and Purpose:**

The optimal management of early-stage follicular lymphoma (FL) remains an active research area, particularly in China. This retrospective cohort study examined long-term survival outcomes, recurrence patterns, and potential prognostic factors in patients with early-stage FL.

**Materials and Methods:**

We retrospectively analyzed 107 patients diagnosed with early-stage FL between 2000 and 2020 at the National Cancer Center in China. Treatment modalities included radiotherapy with or without chemoimmunotherapy or chemotherapy, chemoimmunotherapy or chemotherapy, and observation. Overall survival, progression-free survival, and lymphoma-specific survival were assessed using the Kaplan-Meier method and Cox regression models. We evaluated failure patterns, including locoregional and systemic failures, using cumulative incidence analysis with competing risks. Exploratory LASSO regression and machine learning–based approaches were applied to identify potential prognostic factors.

**Results:**

The median age was 53 years, and the median follow-up was 86 months. In this 20-year real-world cohort from the National Cancer Center of China (n = 107), long-term outcomes were favorable, with 5- and 10-year rates of 88.7% and 68.8% for OS, 93.3% and 89.9% for LSS, and 76.0% and 57.2% for PFS. No statistically significant differences in survival or cumulative failure were observed across initial management strategies, including radiotherapy with or without systemic therapy, systemic therapy alone, and observation. Recurrence occurred in 21 patients (19.6%), predominantly locoregional (14 locoregional only vs. 6 systemic only; 1 both). The 5- and 10-year cumulative incidences were 16.3% and 19.5% for overall failure, 14.5% and 16.2% for locoregional failure, and 3.1% and 4.8% for systemic failure. POD24 occurred in 6.5% of patients and was associated with inferior OS, although event numbers were limited. Low neutrophil-to-lymphocyte ratio (NLR <1.8) was associated with poorer survival in exploratory analyses.

**Conclusion:**

Patients with early-stage FL in this 20-year real-world Chinese cohort demonstrated an overall favorable long-term prognosis across diverse initial management strategies. No treatment modality showed a clear survival advantage in this limited-size cohort, while relapse was uncommon and more frequently locoregional than systemic. POD24 identified a small subset of patients with inferior survival, highlighting the need for individualized, risk-adapted management and long-term follow-up.

## Introduction

Follicular lymphoma (FL) is an indolent hematological malignancy. Early-stage disease (Ann Arbor stages I–II) accounts for approximately 20%–30% of all cases (Freedman and Jacobsen, 2020; Yang and Yahalom, 2018; Pugh et al., 2010). FL is more prevalent in Western populations (Cerhan, 2020; Shirley et al., 2013) than in Asian countries. In China, FL constitutes only 5%–10% of all non-Hodgkin lymphoma cases (Cao et al., 2018; Meng et al., 2018). Despite this lower incidence, contemporary epidemiologic and clinical data from Asian populations—particularly regarding early-stage FL—remain limited, underscoring the need to better characterize real-world management patterns and outcomes in this setting.

Although the interest in FL is growing, much recent research has focused on advanced-stage disease (Ann Arbor stages III–IV) (Fowler et al., 2022; Kim et al., 2024; McClanahan et al., 2010). While these studies offer valuable insights into treatment strategies and survival benefits, their applicability to early-stage disease is limited due to differences in tumor biology, treatment responses, and clinical management (Kridel 2025). For early-stage disease, several large Western prospective trials and real-world analyses have provided comprehensive descriptions of survival outcomes and relapse patterns (Brady et al., 2018; Tobin et al., 2019; Sorigue et al., 2018; Hoskin et al., 2021). However, these data derive almost exclusively from Western populations, and comparable long-term evaluations from Chinese cohorts are largely absent. As a result, the patterns of locoregional and systemic failure, their prognostic implications, and the competing risks of lymphoma-related versus non–lymphoma-related mortality remain insufficiently characterized in China, despite their relevance for treatment decision-making. These gaps limit the ability to generalize findings from Western trials to regions where disease prevalence, diagnostic practices, and treatment availability may differ.

Furthermore, while radiotherapy (RT) has consistently demonstrated excellent local control in early-stage FL (Lo et al., 2020; Guadagnolo et al., 2006; Sha et al., 2022), the real-world recurrence spectrum across different initial management strategies—including RT, systemic therapy, and observation—has not been systematically evaluated in Chinese patients. Over the past 2 decades, changes in diagnostic workflows—particularly increasing use of FDG-PET/CT—have also influenced staging accuracy and treatment selection, making contemporary longitudinal data particularly valuable for understanding clinical outcomes across different eras of practice. In addition, the cumulative incidence of disease failure and mortality, and the prognostic value of clinical or hematologic factors, have not been well characterized in Chinese early-stage FL—despite their relevance for personalized risk assessment and treatment planning.

To address these critical gaps, we conducted a 20-year retrospective study at the National Cancer Center of China. By integrating detailed survival analysis, competing-risk evaluation of locoregional and systemic failure, and exploratory assessment of prognostic factors this study provides a comprehensive real-world characterization of early-stage FL in a Chinese population.

## Materials and Methods

### Data source and study population

We conducted a retrospective analysis on patients diagnosed with early-stage FL, grades 1–3A, at the National Cancer Center, Cancer Hospital, Chinese Academy of Medical Sciences, Peking Union Medical College between 2000 and 2020. The inclusion criteria were: (1) histologically confirmed grade 1–3A FL (Alaggio et al., 2022); (2) Ann Arbor stage I–II disease; and (3) age ≥18 years at the time of diagnosis. The exclusion criteria were: (1) Ann Arbor stage III–IV disease; (2) incomplete data on primary treatment; and (3) loss to follow-up within 3 months after diagnosis. The study included 107 patients with early-stage FL. Bulky disease was defined as a nodal or extranodal mass with the largest diameter exceeding 5 cm. Because the study period spanned 2 decades, staging procedures evolved over time. PET/CT was performed in 31.8% of patients overall, with lower utilization in the earlier years and increasing adoption after 2010. All patients underwent bone marrow biopsy as part of baseline staging. Marrow flow cytometry was not routinely performed during the study period. For patients without PET/CT, staging was determined using contrast-enhanced CT, clinical evaluation, and bone marrow biopsy findings. Ann Arbor stage I–II was assigned based on the best available imaging and pathological information at diagnosis. This study was conducted in accordance with the Declaration of Helsinki and approved by the PUMC/CAMS independent ethics committee (no. 25/164-5110). Given the retrospective nature of the study and the use of de-identified data, the requirement for informed consent was waived by the ethics committee.

### Treatments

The first-line treatment strategy included RT with or without chemoimmunotherapy or chemotherapy (RT ± CIT, *n* = 57), chemoimmunotherapy or chemotherapy (CIT, *n* = 34), and observation (*n* = 16). The RT ± CIT category included patients treated with radiotherapy alone or with radiotherapy combined with chemotherapy or chemoimmunotherapy, whereas the CIT group consisted of patients receiving chemotherapy or chemoimmunotherapy without radiotherapy. Observation was defined as a documented decision in the medical record to defer treatment for at least 3 months from the date of diagnosis, with no treatment during this period. The treatment decision was at the discretion of the physician or the patient’s choice. Treatment response was primarily assessed using the Lugano criteria (Cheson et al., 2014).

The RT ± CIT group received involved-field or involved-site RT. The median dose was 30 Gy (interquartile range [IQR], 30–40 Gy), at 1.8–2.0 Gy per fraction. Most patients received intensity-modulated or three-dimensional conformal RT. The specific dose fractionation schedules and RT techniques are shown in Supplementary Table S1.

Systemic therapy regimens reflected real-world management of follicular lymphoma across the two-decade study period. For analytical purposes, systemic therapy was defined as the administration of chemotherapy and/or immunotherapy without radiotherapy, or as part of combined-modality treatment combined with radiotherapy. Regimens were classified according to their dominant components and included anthracycline-based chemoimmunotherapy (e.g., CHOP or R-CHOP–based), chemotherapy alone, rituximab-based immunotherapy without cytotoxic agents, and other less commonly used regimens such as lenalidomide-containing or alkylator-based therapies. Treatment selection was individualized based on patient age, comorbidities, performance status, and disease characteristics, consistent with real-world practice during the study period.

To more comprehensively evaluate differences across therapeutic strategies, we additionally applied a four-category grouping—radiotherapy alone (RT), systemic therapy alone (CIT), combined systemic therapy plus radiotherapy (CIT + RT), and observation—in exploratory analyses. This treatment-modality–based grouping provided clinical granularity for comparing specific approaches, while the primary three-group framework supported coherent statistical modeling and interpretation. The exploratory categorization was not used for primary endpoints but served to complement and contextualize treatment-related findings.

### Endpoints

The primary endpoints of this study were overall survival (OS), lymphoma-specific survival (LSS), and PFS. OS was defined as the time from diagnosis to death from any cause. LSS was defined as the time from diagnosis to death due to FL. PFS was defined as the time from diagnosis to disease progression, relapse, or death from any cause. Non-lymphoma-related death (non-LRD) was defined as death due to causes other than FL, including cardiovascular disease, secondary malignancies, and other non-lymphoma conditions. Disease failure was defined as the recurrence or progression of FL after initial treatment. Progression of disease within 24 months (POD24) was defined as disease progression, relapse, transformation, or death from any cause occurring within 24 months of the initiation of first active anti-lymphoma therapy. For patients who received immediate treatment after diagnosis, the 24-month interval was measured from the start of first-line therapy. For patients initially managed with observation, the interval was measured from the initiation of their first systemic therapy or radiotherapy. Patients who remained untreated and alive beyond 24 months from diagnosis were classified as non-POD24, consistent with definitions used in prior early-stage and real-world FL studies. Locoregional failure (LRF) was defined as the recurrence or progression of FL within the original tumor site or regional lymph nodes. Systemic recurrence of FL was defined as systemic disease progression or recurrence at sites distinct from the originally involved sites, including distant lymph nodes or extranodal locations.

### Statistical analysis

Categorical variables were compared using the chi-squared or Fisher’s exact test. Survival outcomes were estimated using the Kaplan-Meier method, and survival differences were compared by the log-rank test. The Fine-Gray competing risk model was applied to evaluate the cumulative incidence of lymphoma-specific mortality and non-LRD, accounting for competing risks. Multivariable analysis was performed using Cox proportional hazards models to identify independent prognostic factors for OS and PFS, while competing risk regression was used for LSS and non-LRD. LASSO regression with ten-fold cross-validation was performed using the *glmnet* package in R to identify prognostic variables associated with overall survival from 24 clinical and hematologic factors (*Sex, Age, Year, Stage, Primary site, Grade, Ki-67, B symptoms, FLIPI1, FLIPI2, Bulky disease, Lymph involvement, ECOG, LDH, Leukopenia, Anemia, PLT, Lymphopenia, NLR, RT, PFS24, CT, Surgery,* and *CD20*). Variables with nonzero coefficients at the optimal penalty parameter (λ) were selected for model construction. Three survival prediction models were subsequently developed, including a Cox proportional hazards model, a Gradient Boosting Machine (GBM) model, and a Random Survival Forest (RSF) model. Model performance was evaluated using the concordance index (C-index) and time-dependent receiver operating characteristic (ROC) curves at 3-, 5-, and 10-year time horizons. The RSF model demonstrated the best predictive discrimination and was further interpreted using SHAP (SHapley Additive exPlanations) analysis to quantify the global contribution and relative importance of each feature across different prediction horizons. Statistical analysis was conducted using R software (version 4.3.3), and a *P*-value <0.05 was considered statistically significant.

## Results

### Baseline patient characteristics

The baseline patient characteristics are shown in Table 1. The median age at diagnosis was 53 years (IQR, 44–62), with 30.8% of patients under the age of 60. Most patients (98.1%) had an Eastern Cooperative Oncology Group (ECOG) performance status of 0–1, and 57.9% presented with a stage II disease. Histologically, 61.7% of patients had grade 1–2 FL, 29.0% had grade 3A disease, while grade information was not specified for the remaining 9.3% of patients. Bulky disease was observed in 11.2% of patients. Hematologic abnormalities were observed at diagnosis in some patients, including leukopenia (13.1%), anemia (2.8%), lymphopenia (4.7%), and thrombocytopenia (1.9%). Elevated lactate dehydrogenase (LDH ≥245 U/L) was found in 8.4% of patients, and 36.4% had elevated β2-microglobulin (≥3 mg/L). The neutrophil-to-lymphocyte ratio (NLR) was <1.8 in 30.8% of patients. Furthermore, 63.5% of patients were diagnosed between 2015 and 2020, which suggests a recent increase in early-stage FL diagnoses.

**TABLE 1:** Baseline characteristics of early-stage FL patients stratified by primary treatment.

Characteristic	Overall	Treatment			
		RT ± CIT	CIT	Observation	​
	No. (%)	No. (%)	No. (%)	No. (%)	*P*
Number of patients	107	57	34	16	​
Gender, male	57 (53.3)	28 (49.1)	22 (64.7)	7 (43.8)	0.255
Age (years)	​	​	​	​	0.110
Median (range)	53 (27–87)	53 (27–87)	56 (34–85)	53 (37–67)	​
≤60	74 (69.2)	42 (73.7)	19 (55.9)	13 (81.3)	​
ECOG performance status	​	​	​	​	0.772
0–1	105 (98.1)	56 (98.2)	33 (97.1)	16 (100)	​
2–4	2 (1.9)	1 (1.8)	1 (2.9)	0 (0)	​
Ann arbor stage	​	​	​	​	0.134
I	45 (42.1)	24 (42.1)	11 (32.4)	10 (62.5)	​
II	62 (57.9)	33 (57.9)	23 (67.6)	6 (37.5)	​
Histological grade	​	​	​	​	0.375
I-II	66 (61.7)	38 (66.7)	18 (53.0)	8 (62.4)	​
IIIA	31 (29.0)	18 (31.6)	10 (29.4)	3 (18.8)	​
Unspecified	10 (9.3)	1 (1.8)	6 (17.6)	3 (18.8)	​
Ki-67 index	​	​	​	​	0.283
<20%	17 (15.9)	12 (21.1)	3 (8.8)	2 (12.5)	​
≥20%	90 (84.1)	45 (78.9)	31 (91.2)	14 (87.5)	​
B Symptoms	8 (7.5)	5 (8.8)	3 (8.8)	0 (0)	0.317
Primary site	​	​	​	​	0.412
Supradiaphragmatic nodes	51 (47.7)	28 (49.1)	17 (50.0)	6 (37.5)	​
Subdiaphragmatic nodes	33 (30.8)	18 (31.6)	11 (32.4)	4 (25.0)	​
Extranodal	23 (21.5)	11 (19.3)	6 (17.6)	6 (37.5)	​
FLIPI score (low-risk, 0–1)	97 (90.7)	52 (91.2)	29 (85.3)	16 (100)	0.541
FLIPI2 score (low-risk, 0–1)	100 (93.5)	56 (98.2)	29 (85.3)	15 (93.8)	0.054
Bulky disease ≥ 5 cm	12 (11.2)	8 (14.0)	4 (11.8)	0 (0)	0.292
No. of involved lymph nodes	​	​	​	​	0.737
0–1	63 (58.9)	35 (61.4)	17 (50.0)	11 (68.8)	​
2–3	34 (31.8)	18 (31.6)	11 (32.4)	5 (31.3)	​
≥4	10 (9.3)	4 (7.0)	6 (17.6)	0 (0)	​
Hematologic abnormality					
Anemia	3 (2.8)	2 (3.5)	1 (2.9)	0 (0)	0.755
Leukopenia	14 (13.1)	9 (15.8)	5 (14.7)	0 (0)	0.243
Lymphopenia	5 (4.7)	3 (5.3)	2 (5.9)	0 (0)	0.628
Thrombocytopenia	2 (1.9)	2 (3.5)	0 (0)	0 (0)	0.412
Elevated LDH	9 (8.4)	4 (7.0)	5 (14.7)	0 (0)	0.086
β2-MG ≥ 3 mg/L	​	​	​	​	0.002*
Yes	39 (36.4)	26 (45.6)	12 (35.3)	1 (6.3)	​
No	56 (52.4)	29 (50.9)	18 (52.9)	9 (56.3)	​
Missing data	12 (11.2)	2 (3.5)	4 (11.8)	6 (37.5)	​
NLR	​	​	​	​	0.008*
<1.8	33 (30.8)	25 (43.9)	6 (17.6)	2 (12.5)	​
≥1.8	74 (69.2)	32 (56.1)	28 (82.4)	14 (87.5)	​

Abbreviations: RT±CIT, radiotherapy with or without chemoimmunotherapy or chemotherapy; CIT, chemoimmunotherapy or chemotherapy; ECOG PS, eastern cooperative oncology group performance status; FLIPI, follicular lymphoma international prognostic index; LDH, lactate dehydrogenase; β2-MG, Beta-2 microglobulin; NLR, Neutrophil-to-Lymphocyte Ratio. Bold values and asterisks (*) indicate statistical significance (p < 0.05).

Among the 72 patients who received systemic therapy (either alone or in combination with radiotherapy), treatment patterns reflected contemporary real-world practice. R-CHOP–based chemoimmunotherapy was the most common regimen (49 patients, 68.1%), followed by CHOP alone (14 patients, 19.4%). Rituximab monotherapy was administered in 7 patients (9.7%), and lenalidomide-based regimens, including R^2^-like combinations, were used in 4 patients (5.6%).

### Causes of death and survival stratified by treatment

**FIGURE 1:**
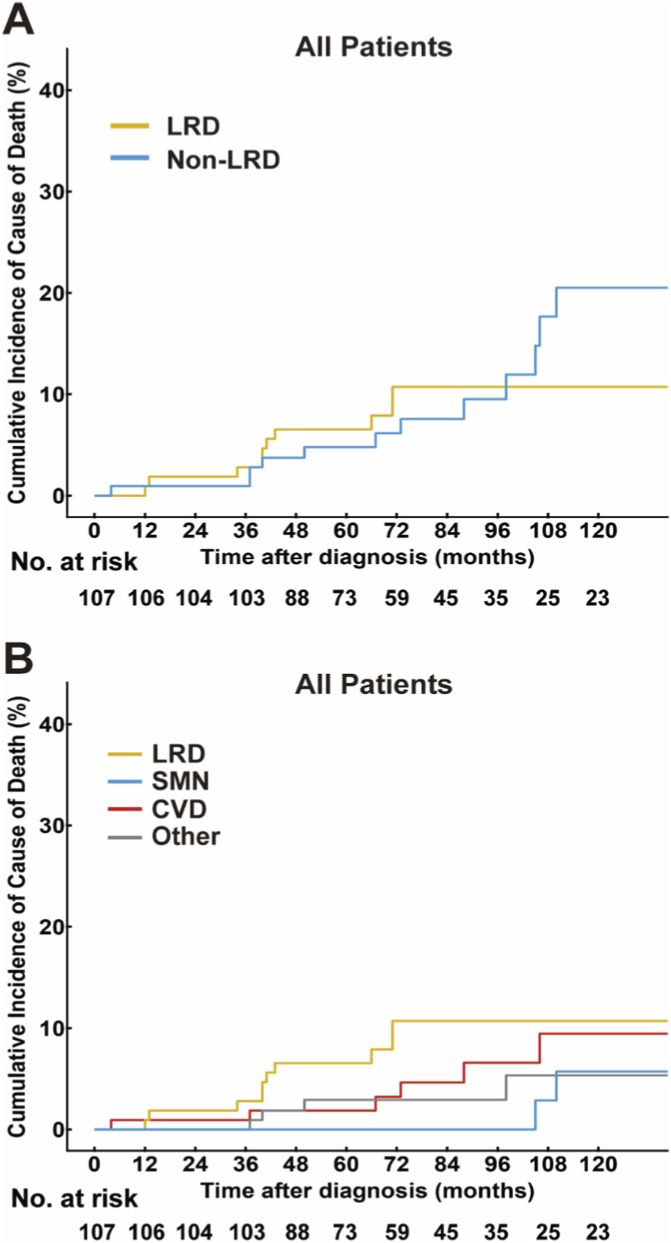
Cause of death in patients with early-stage FL. **(A)** Cumulative incidences of LRD, non-LRD, and all deaths; **(B)** Cumulative incidences of SMN and other diseases besides FL. Abbreviations: LRD, lymphoma related death; non-LRD, non-lymphoma related death; SMN, secondary malignant neoplasms; FL, follicular lymphoma.

Twenty-three patients died during a median follow-up of 86 months (IQR, 59–144), including LRD in 10 patients, and non-LRD in 13. Using non-LRD as a competing risk, the cumulative incidence of LRD slightly increased from 6.5% at 5 years (95% confidence interval [CI]: 2.9%–12.3%) to 10.7% at 10 years (95% CI: 5.4%–18.2%). Conversely, the cumulative incidence of non-LRD markedly increased from 4.8% at 5 years (95% CI: 1.8%–10.1%) to 20.5% at 10 years (95% CI: 10.3%–33.3%; Figure 1A). Among the 13 non-LRD events, cardiovascular disease was the most common cause (6/13, 46.2%), followed by other non–cancer-related medical conditions (4/13, 30.8%) and secondary malignant neoplasms (3/13, 23.1%; rectal, breast, and laryngeal cancers), as shown in Figure 1B.

### Failure pattern and cumulative incidence

**FIGURE 2:**
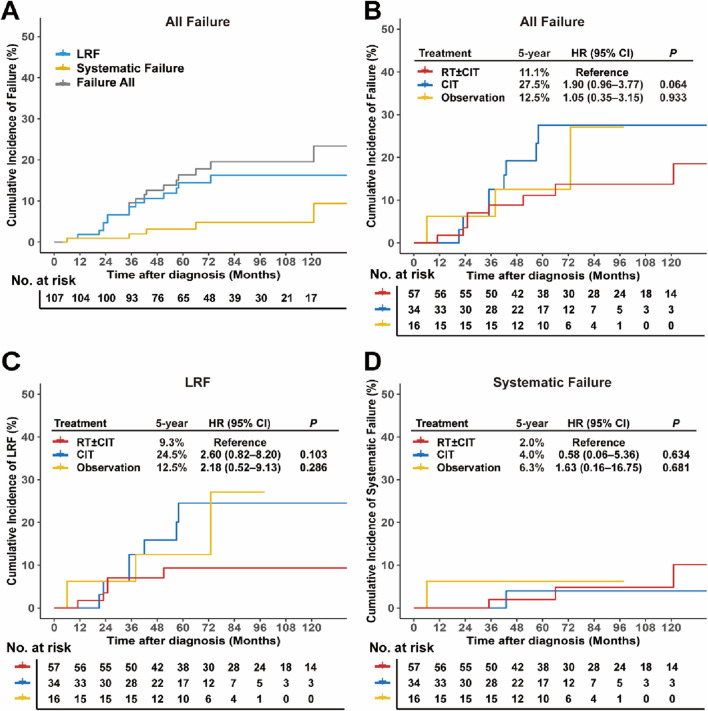
Cumulative incidence of failure patterns in early-stage FL stratified by treatment strategies. **(A)** Cumulative incidence curves showing all failures, LRF, and systemic failures among all patients. Cumulative incidence of **(B)** all failures, **(C)** LRF, and **(D)** systemic failures stratified by treatment strategies. Abbreviations: FL, follicular lymphoma; LRF, locoregional failure.

Twenty-one patients (19.6%) developed disease relapse by the last follow-up. Fourteen patients developed LRF only (five with local failure only, six with regional lymph node failure only, and three with both), six had systemic failure only, and one had both LRF and systemic failures. The 5-year and 10-year cumulative incidences were 14.5% and 16.2% for LRF, 3.1% and 4.8% for systemic failure, and 16.3% and 19.5% for overall failures (Figure 2A).

In most patients, failure occurred early in the course of the disease. The disease had relapsed within 5 years in 16 patients and after more than 5 years in five patients. No significant differences in failure rates were observed among the treatment modality groups. The 5-year cumulative incidences of overall failure were 11.1% for RT ± CIT, 27.5% for CIT (hazard ratio [HR], 1.90; 95% CI: 0.96–3.77; *P* = 0.064; Figure 2B), and 12.5% for observation (HR, 1.05; 95% CI: 0.35–3.15; *P* = 0.933; Figure 2B). The corresponding 10-year cumulative incidences were 18.5%, 27.5%, and unreached.

For LRF, the 5-year cumulative incidences were 9.3% for RT ± CIT, 24.5% for CIT (HR, 2.60, 95% CI: 0.82–8.20; *P* = 0.103; Figure 2C), and 12.5% for observation (HR, 2.18; 95% CI: 0.52–9.13; *P* = 0.286; Figure 2C). The respective 10-year rates were 9.3%, 24.5%, and unreached. Systemic failure was rare across all groups, with 5-year cumulative incidences of 2.0% for RT ± CIT, 4.0% for CIT (HR, 0.58; 95% CI: 0.06–5.36; *P* = 0.634; Figure 2D), and 6.3% for observation (HR, 1.63; 95% CI: 0.16–16.75; *P* = 0.681; Figure 2D). The corresponding 10-year incidences were 10.1%, 4.0%, and unreached.

Among the patients in the RT ± CIT group, ten experienced failures, including five in-field failures (within the composite 95% isodose volume) and five out-of-field failures (outside the irradiated volume). The time to in-field recurrences ranged between 9.9 and 50.9 months. Out-of-field failures primarily involved nodal relapses outside the irradiated field. A detailed breakdown of in-field recurrence patterns, including irradiated volumes and doses, is presented in Supplementary Table S2. To further evaluate whether treatment granularity affected failure patterns, we conducted an exploratory four-group analysis separating RT alone, CIT, CIT + RT, and observation (Supplementary Figure S1). Although not powered for formal statistical comparison due to limited event numbers within individual strata, the cumulative incidence curves for overall failure, locoregional failure, and systemic failure showed broadly similar trajectories across all four categories. No clinically meaningful divergence was observed.

### Survival stratified by treatment

**FIGURE 3:**
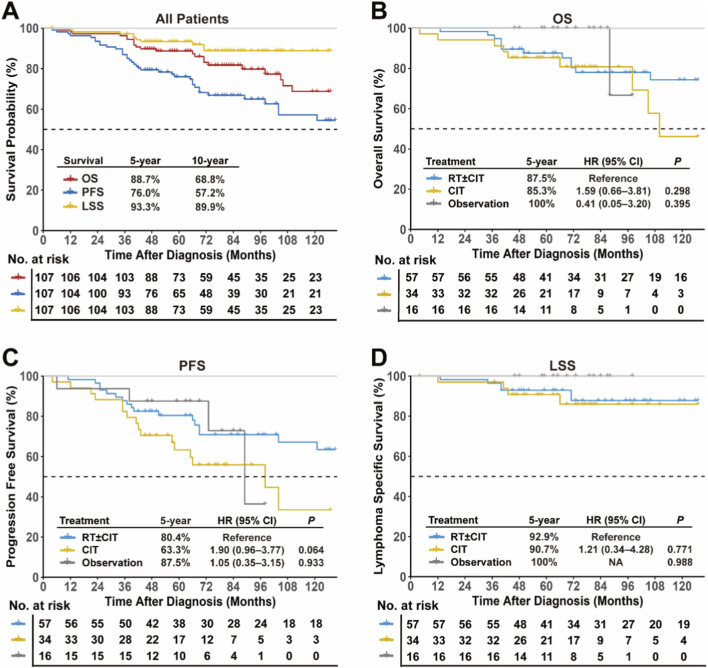
Survival outcomes in early-stage FL stratified by treatment strategies. **(A)** Kaplan-Meier curves for OS, PFS, and LSS among all patients. Comparison of **(B)** OS, **(C)** PFS, and **(D)** LSS across treatment groups. Abbreviations: FL, follicular lymphoma; OS, overall survival; PFS, progression-free survival; LSS, lymphoma-specific survival.

The 5- and 10-year rates for the entire cohort were 88.7% and 68.8% for OS (95% CI: 82.8%–94.9%), 93.3% and 89.9% for LSS (95% CI: 88.2%–94.7%; Figure 3A), and 76.0% and 57.2% for PFS (95% CI: 68.2%–84.7%). POD24 occurred in 6.5% of patients.

There were no statistically significant differences in survival between treatment groups. The 5-year OS, PFS, and LSS rates were 87.5%, 80.4%, and 92.9% in the RT ± CIT group; 85.3% (HR, 1.59; 95% CI: 0.66–3.81; *P* = 0.295; Figure 3B), 63.3% (HR, 1.90; 95% CI: 0.96–3.77; *P* = 0.064; Figure 3C), and 90.7% (HR, 1.21; 95% CI: 0.34–4.28; *P* = 0.771; Figure 3D) in the CIT group; and 100% (HR, 0.41; 95% CI: 0.05–3.20; *P* = 0.395; Figure 3B), 87.5% (HR, 1.05; 95% CI: 0.35–3.15; *P* = 0.933; Figure 3C), and 100% (HR, not available; *P* = 0.988; Figure 3D) in the observation group.

To complement the primary treatment-group comparison, an exploratory four-category survival analysis was also performed (RT alone, systemic therapy alone, systemic therapy plus RT, and observation; Supplementary Figure S2). As expected with small sample sizes and few survival events, the curves did not demonstrate statistically or clinically significant separation. The overall patterns remained consistent with the main analysis, indicating that the absence of major survival differences is not dependent on the specific grouping scheme.

### Failure patterns and their association with survival

We analyzed failure within a competing risk framework, considering non-LRD as a competing event. As shown in (Supplementary Figure S3A), the cumulative incidence of failure increased steadily over time, surpassing that of non-LRD after 24 months. Stratification by treatment modality revealed no statistically significant differences in failure risk (Supplementary Figure S3B) or the risk of non-LRD (Supplementary Figure S3C) among groups.

Assessment of OS by failure incidence showed 5-year OS rates of 89.5% without failure, 83.9% with LRF and 87.5% with systemic failure, with no significant difference between groups (Supplementary Figure S4A). Patients who experienced POD24 exhibited significantly poorer OS than those those who did not experience POD24 (*P* = 0.011; Supplementary Figure S4B). Cumulative incidence analysis further confirmed that patients who experienced POD24 had a significantly higher incidence of treatment failure and death than those who did not experience POD24, underscoring the critical prognostic impact of early disease progression (Table 2).

**TABLE 2:** Cumulative incidence of disease failure and mortality.

Characteristics	Failure (%)			Death (%)		
	5-year	10-year	*P*	5-year	10-year	*P*
All patients	16.3	19.5	-	11.3	31.3	-
Gender	​	​	0.025*	​	​	<0.001*
Male	22.7	25.6	​	16.1	37.0	​
Female	9.0	12.4	​	6.0	25.4	​
Age (years)	​	​	0.152	​	​	<0.001*
≤60	14.6	16.6	​	2.7	9.3	​
>60	20.8	29.6	​	31.7	81.3	​
ECOG performance status	​	​	0.174	​	​	0.007*
0–1	15.7	22.8	​	10.5	28.6	​
2–4	50.0	N/A	​	50.0	N/A	​
Ann arbor stage	​	​	0.653	​	​	0.565
I	14.3	14.3	​	7.2	35.6	​
II	17.8	23.1	​	14.5	29.6	​
Histological grade	​	​	0.454	​	​	0.696
I-II	16.3	22.4	​	12.5	37.5	​
IIIA	14.3	14.3	​	12.9	26.5	​
Unspecified but low grade	20.0	20.0	​	0.0	12.5	​
Ki-67 index	​	​	0.874	​	​	0.214
<20%	18.8	18.8	​	24.5	35.3	​
≥20%	15.7	19.2	​	8.9	30.3	​
B Symptoms	​	​	0.955	​	​	0.461
Present	14.3	14.3	​	25.0	25.0	​
Absent	16.0	19.6	​	10.7	31.9	​
Missing data	25.0	25.0	​	0.0	0.0	​
Primary site	​	​	0.837	​	​	0.184
Supradiaphragmatic nodes	15.1	22.1	​	13.7	41.8	​
Subdiaphragmatic nodes	20.6	20.6	​	9.1	18.6	​
Extranodal	13.0	13.0	​	9.4	32.0	​
FLIPI score	​	​	0.078	​	​	<0.001*
Low risk (score 0–1)	14.4	17.7	​	5.2	24.3	​
Intermediate and high risk (score 2–3)	42.9	42.9	​	70.0	85.0	​
FLIPI2 score	​	​	0.308	​	​	<0.001*
Low risk (score 0–1)	17.2	20.5	​	10.1	28.9	​
Intermediate and high risk (score 2–3)	N/A	N/A	​	28.6	64.3	​
Bulky disease ≥ 5 cm	​	​	0.600	​	​	0.491
Yes	15.7	19.4	​	0	20.0	​
No	18.2	18.2	​	12.8	33.6	​
No. of involved lymph nodes area	​	​	0.176	​	​	0.481
0–1	15.8	15.8	​	6.6	28.2	​
2–3	13.0	18.8	​	23.5	32.0	​
≥4	30.0	44.0	​	0	57.1	​
Hematologic abnormality						
Anemia	N/A	N/A	-	66.7	66.7	0.023*
Leukopenia	24.5	24.5	0.333	35.7	51.8	0.021*
Lymphopenia	25.0	25.0	0.777	40.0	70.0	0.007*
Thrombocytopenia	50.0	50.0	0.110	50.0	N/A	0.005*
Elevated LDH	41.7	41.7	0.354	33.3	50.0	0.068
β2-MG ≥ 3 mg/L	​	​	0.618	​	​	0.235
Yes	21.5	21.5	​	15.4	40.2	​
No	15.0	20.6	​	10.9	27.5	​
Missing data	8.3	8.3	​	0	0	​
NLR	​	​	0.025*	​	​	0.017*
<1.8	27.0	27.0	​	19.2	51.9	​
≥1.8	11.7	15.7	​	8.1	23.8	​
POD24	​	​	<0.001*	​	​	0.005*
Yes	100	100	​	42.9	42.9	​
No	12.2	15.5	​	9.1	29.7	​

Abbreviations: RT±CIT, radiotherapy combined with chemotherapy or chemoimmunotherapy; CIT, chemotherapy or chemoimmunotherapy; ECOG PS, eastern cooperative oncology group performance status; FLIPI, follicular lymphoma international prognostic index; LDH, lactate dehydrogenase; β2-MG, Beta-2 microglobulin; NLR, Neutrophil-to-Lymphocyte Ratio; POD24, Progression of disease within 24 months. Bold values and asterisks (*) indicate statistical significance (p < 0.05).

### Prognostic factors

Univariate Cox regression analysis identified several significant prognostic factors for mortality in early-stage FL (Table 2). These included age over 60 years (*P* < 0.001), male sex (*P* = 0.007), ECOG score ≥2 (*P* = 0.007), NLR ≥1.8 (*P* = 0.017), anemia (*P* = 0.023), leukopenia (*P* = 0.021), thrombocytopenia (*P* = 0.005), and lymphopenia (*P* = 0.007). Elevated LDH (*P* = 0.068) and β2-microglobulin (*P* = 0.235) lacked statistical significance. Male sex (*P* = 0.026) and NLR <1.8 (*P* = 0.025) were significantly associated with an increased risk of failure.

**FIGURE 4:**
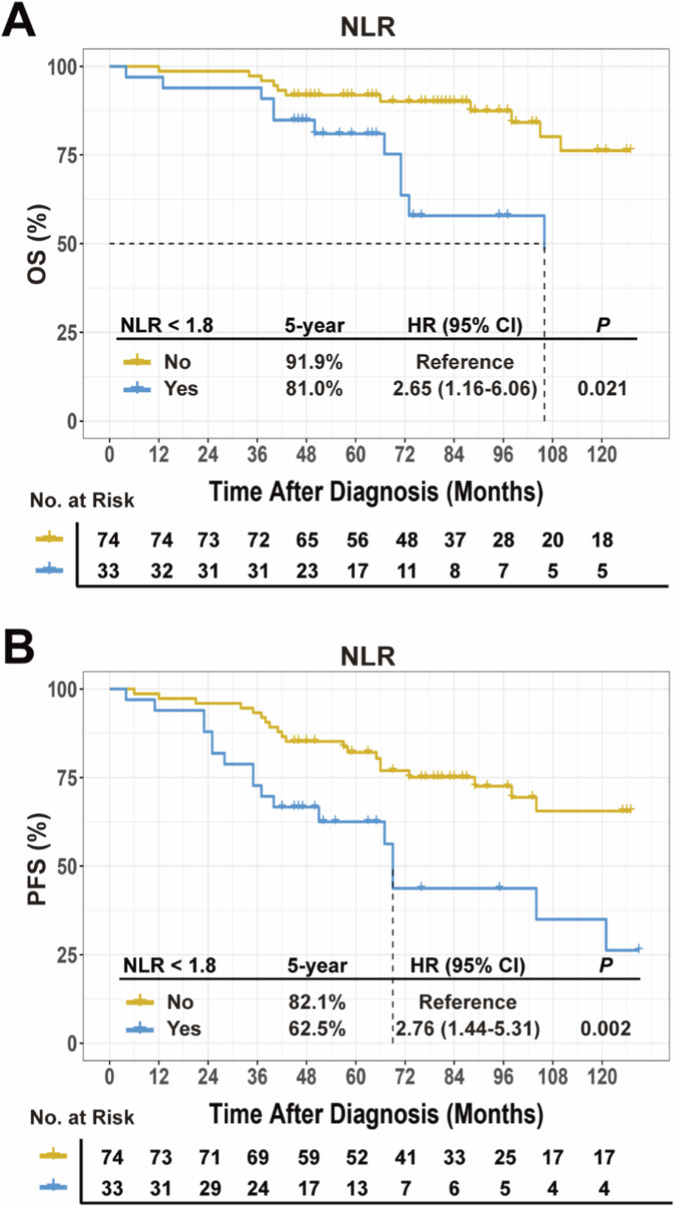
Prognostic association of NLR with **(A)** OS and **(B)** PFS in early-stage FL patients. Abbreviations: NLR, neutrophil-to-lymphocyte ratio; OS, overall survival; PFS, progression free survival; FL, follicular lymphoma.

Kaplan-Meier survival analysis showed that, compared to patients with NLR ≥1.8, those with NLR <1.8 exhibited significantly worse 5-year OS (81.0% vs. 91.9%; HR, 2.65; 95% CI: 1.16–6.06; *P* = 0.021; Figure 4A) and PFS (62.5% vs. 82.1%; HR, 2.76; 95% CI: 1.44–5.31; *P* = 0.002; Figure 4B).

To explore prognostic factors, LASSO regression was used to select survival-associated clinical and laboratory variables (Supplementary Figure S5A,B). These variables were incorporated into three prediction models (Cox, GBM, RSF). The RSF model showed the best discrimination, with a C-index of 0.943 compared with 0.888 for the Cox model and 0.734 for the GBM model. Time-dependent ROC curves consistently demonstrated superior AUCs for the RSF model at 3, 5, and 10 years (Supplementary Figure S5C–E). Model interpretability was assessed using SHAP analysis. Age was the most influential predictor, followed by lymphopenia and FLIPI1, indicating the importance of both demographic and immune-related factors. SHAP heatmaps demonstrated stable feature importance across time horizons (Supplementary Figure S5F,G).

## Discussion

This study provides a comprehensive real-world assessment of early-stage FL within the national cancer center in China, addressing an important evidence gap in Asian populations. Despite differences in practice patterns and access to staging technologies over the 2 decades covered, patients achieved excellent long-term OS and LSS, closely mirroring outcomes reported in contemporary Western cohorts. These findings reinforce the consistently favorable prognosis of limited-stage FL across diverse geographic and clinical settings.

The overall and lymphoma-specific survival observed in this cohort aligns closely with outcomes reported in Western population-based registries, where 5-year OS typically ranges from 70% to 80% and lymphoma-specific survival exceeds 90% [Junlén et al., 2015; Sarkozy et al., 2019; Zhong et al., 2024). Our 5-year PFS (80.3%) was consistent with outcomes observed in prior studies of early-stage FL. The MIR trial, which evaluated RT combined with rituximab, reported similarly favorable disease control (Herfarth et al., 2018), while the Canadian RT-alone series demonstrated high LSS (Campbell et al., 2010). Despite differing treatment strategies and endpoints, these studies collectively reinforce the excellent prognosis of limited-stage FL. Across both the primary three-group classification and the exploratory four-group categorization, survival differences were not statistically significant. Given the small number of events, the study was clearly underpowered to detect modest survival differences, and the absence of observed differences cannot be construed as evidence of equivalence. Larger multicenter studies within Chinese or broader Asian populations will be required to validate treatment-specific survival trends in this distinct epidemiologic setting.

Recurrence patterns in this cohort provide additional insights into the behavior of limited-stage FL. Locoregional failures were more common than systemic relapses, but this finding should be interpreted in the context of evolving diagnostic and therapeutic practices across the two-decade study period. PET/CT was increasingly used only in later years (Zanoni et al., 2022), and earlier staging relied primarily on CT and bone marrow biopsy, which may have led to under-staging and misclassification of occult systemic disease as isolated locoregional relapse. Radiotherapy practice evolved substantially over the two-decade study period (Yahalom 2014). In earlier years, treatment was commonly delivered using two-dimensional or three-dimensional conformal techniques with relatively large involved-field radiotherapy (IFRT) volumes and higher prescribed doses, in some cases exceeding 40–45 Gy. With advances in imaging, treatment planning, and delivery, radiotherapy gradually transitioned toward more conformal three-dimensional and intensity-modulated approaches, accompanied by a shift from IFRT to involved-site radiotherapy (ISRT). In parallel, prescribed doses converged toward the contemporary standard of approximately 24 Gy (Hoskin et al., 2021), consistent with modern practice guidelines, while more recent phase II studies have explored ultra–low-dose regimens such as 12 Gy in selected indolent lymphoma settings (Wang et al., 2025). These temporal changes in technique, target delineation, and dose selection likely contributed to treatment heterogeneity and may have influenced the observed patterns of in-field and out-of-field failure in this cohort. Therefore, while LRF appeared numerically more common than systemic recurrence in this cohort, this pattern should be viewed within its methodological constraints and not interpreted as evidence that intensifying locoregional approaches would improve survival—particularly given that OS remained excellent and comparable across treatment modalities.

In this cohort, early recurrence, including POD24, was associated with inferior survival. However, the number of POD24 events was very small, and definitions for patients beginning with observation introduce additional uncertainty. As such, although the signal aligns with findings from advanced-stage FL and other lymphomas (MacManus et al., 2018; van der Galiën et al., 2019; Casulo et al., 2022; Leonard 2022; Yang et al., 2021; Zhu et al., 2020), the prognostic relevance of POD24 in early-stage FL requires cautious interpretation and cannot be considered established based on current data.

The prognostic analyses identified several clinical factors associated with inferior OS or PFS, including age, ECOG ≥2, and cytopenias. Interestingly, a low NLR (<1.8) was associated with poorer outcomes (Yang et al., 2020; Sorigue et al., 2018) suggesting that impaired lymphocyte-mediated immune surveillance may play a more substantial role in indolent FL biology. Typical prognostic markers in FL, such as elevated LDH and β2-microglobulin (Relander et al., 2010), were not significantly associated with outcomes in this cohort. This finding may reflect the limited number of events, the relatively favorable risk distribution of early-stage disease, or insufficient statistical power, rather than the absence of prognostic relevance, and warrants cautious interpretation.

An important finding of this study is that non–lymphoma-related mortality ultimately exceeded lymphoma-related mortality with long-term follow-up. This observation has important clinical implications: in a disease characterized by excellent lymphoma-specific survival and a prolonged natural history, the potential toxicity associated with systemic therapy—including the cardiotoxic and immunosuppressive effects of anthracycline- or bendamustine-based regimens—should be carefully balanced against expected clinical benefit. This consideration is particularly relevant in patients with favorable-risk early-stage FL, for whom durable disease control can be achieved with a range of treatment approaches, including localized strategies delivered with contemporary techniques. Collectively, these findings underscore the importance of individualized treatment selection and highlight the need to avoid overtreatment in a population with substantial competing risks of non–lymphoma-related mortality.

This study has several limitations. The retrospective single-center design introduces unavoidable selection bias; staging and treatment differed across eras; sample size restricted statistical power; event counts were low; and salvage strategies could not be fully evaluated. Despite these limitations, the study offers one of the most detailed and long-term characterizations of early-stage FL within an Asian population, providing clinically meaningful insights into survival, recurrence dynamics, and host-related prognostic factors.

In conclusion, patients with early-stage FL in this real-world Chinese cohort achieved excellent long-term survival across diverse management strategies. Most relapses occurred early and were predominantly locoregional, though this pattern must be interpreted within the context of evolving imaging and radiotherapy practices. The superiority of any specific modality could not be established in this underpowered cohort, and the predominance of non-lymphoma-related mortality underscores the importance of avoiding unnecessary treatment toxicity. Early recurrence and host immune characteristics, such as low NLR, may signal higher-risk biology and warrant further study. Collectively, these findings highlight the need for tailored treatment strategies and long-term survivorship care in early-stage FL, particularly within Asian populations where contemporary data remain limited.

## Data Availability

The raw data supporting the conclusions of this article will be made available by the authors, without undue reservation.
