# Supplementary material for: Failure patterns and clinical indications in early-stage follicular lymphoma: a study from the National Cancer Center in China
Source: Front Pharmacol. 2026 Jan 14;17:1745000. doi: 10.3389/fphar.2026.1745000 (PMC12847288; doi:10.3389/fphar.2026.1745000)
Supplement: Supplementary file 1 [file Supplementaryfile1.docx]

**Table S1. Dose and fraction schedule of radiotherapy**

|  | Number of patients (%) |
| --- | --- |
| All | 57 (100) |
| Total dose |  |
| <24 Gy | 2 (3.5) |
| 24 Gy | 11 (19.3) |
| 30 Gy | 19 (33.3) |
| 36 Gy | 8 (14.0) |
| 40 Gy | 10 (17.5) |
| 45 Gy | 7 (12.3) |
| Dose per fraction |  |
| 1.8 Gy | 8 (14.0) |
| 2.0 Gy | 46 (80.7) |
| Other | 3 (5.3) |
| Radiotherapy technology |  |
| IMRT | 16 (28.1) |
| VMAT | 27 (47.4) |
| TOMO | 1 (1.8) |
| Electron | 4 (7.0) |
| Other | 9 (15.8) |

**Abbreviations:** Gy, gray; IMRT, intensity-modulated radiotherapy; VMAT, volume modulated arc therapy; TOMO, Tomotherapy; Electron, extensive electron beam therapy.

**Table S2. Patterns of in-field recurrence in patients receiving radiotherapy.**

|  | Primary Site Location | CTV Range | Radiotherapy techniques | Radiation Dose (Gy) | Patterns of In-Field Recurrence | Time to recurrence after RT (months) |
| --- | --- | --- | --- | --- | --- | --- |
| 1 | Left Tonsil, Left Cervical Lymph Nodes | The left tonsil and the left cervical lymphatic drainage area | 2D-RT | 45 | The primary tumor of the left tonsil has enlarged in its original location. | 9.9 |
| 2 | Bilateral Parotid Glands, Left Cervical Lymph Nodes | The CTV is delineated by expanding the GTV by 0.5 cm in all three dimensions and includes the bilateral carotid sheaths, bilateral lymphatic drainage regions of levels Ib, II, III, IV, Va, and most of level Vb, as well as the bilateral supraclavicular lymphatic drainage regions and the upper mediastinal lymphatic drainage regions (including the presternal region above the sternum, mediastinal levels 2, 4, 3a, and part of level 5). | IMRT | 40 | Enlargement of cervical lymph nodes within the target region | 50.9 |
| 3 | Nasopharynx, Oropharynx, Bilateral Cervical Lymph Nodes | The CTV encompasses the nasopharynx, oropharynx, lateral pharyngeal walls, base of the tongue, bilateral cervical lymphatic drainage regions of levels Ib, II, and III, as well as the lymphatic drainage regions of levels IV and V, including the supraclavicular and infraclavicular lymph nodes. | IMRT | 40 | Enlargement of cervical lymph nodes within the target region, with newly developed axillary and inguinal lymph node metastases. | 29.7 |
| 4 | Left Inner Thigh Mass | The CTV includes the tumor bed and the inguinal lymphatic drainage regions. | IMRT | 40 | Recurrence of the tumor in the left thigh. | 10.5 |
| 5 | Mesenteric, Retroperitoneal and Para-aortic Lymph Nodes | The CTV includes the pre-chemotherapy mesenteric region, retroperitoneum, intra-abdominal area, posterior pancreatic region, para-aortic cancerous lesions, and high-risk lymphatic drainage areas. | TOMO | 45 | Metastases to mesenteric root lymph nodes, retroperitoneal perivascular lymph nodes, and inguinal lymph nodes. | 14.5 |

**Abbreviations:** CTV, clinical target volume; GTV, gross tumor volume; 2D-RT, two-dimensional radiotherapy; IMRT, intensity-modulated radiotherapy; TOMO, Tomotherapy.

**Supplemental Figures**

**Figure S1.** **Cumulative Incidence of Failure by Four Treatment Categories.** (A) all failure; (B) locoregional failure; and (C) systemic failure stratified by four treatment categories: radiotherapy alone (RT), systemic therapy alone (CIT), systemic therapy combined with radiotherapy (CIT+RT), and observation.

**Figure S2.** **Survival Outcomes by Four Treatment Categories.** (A)overall survival (OS); (B) progression-free survival (PFS), and (C) lymphoma-specific survival (LSS) among patients stratified by four treatment categories: radiotherapy alone (RT), systemic therapy alone (CIT), systemic therapy combined with radiotherapy (CIT+RT), and observation.

**Figure S3. Cumulative incidence of failure and non-LRD across treatment groups.** (A) Cumulative incidence of failure; (B) Cumulative incidence of non-LRD.

Abbreviation: Non-LRD, non-lymphoma-related death.

**Figure S4. Association of failure patterns and POD24 status with OS in early-stage FL.** (A) OS stratified by failure patterns; (B) OS stratified by POD24.

Abbreviations: FL, follicular lymphoma; OS, overall survival; POD24, progression of disease within 24 months.

**Figure S5. Feature selection, model performance, and interpretation of machine learning models.** (A–B) LASSO regression for feature selection showing coefficient profiles and ten-fold cross-validation to determine the optimal λ. (C–E) Time-dependent ROC curves of the Cox, GBM, and RSF models with AUCs at 3, 5, and 10 years. (F–G) Global interpretation of the RSF model using SHAP analysis showing feature importance ranking and stability across different prediction horizons.
Abbreviations: LASSO, least absolute shrinkage and selection operator; ROC, receiver operating characteristic; AUC, area under the curve; GBM, gradient boosting machine; RSF, random survival forest; SHAP, SHapley Additive exPlanations.

**Figure**

**Figure S1**

**
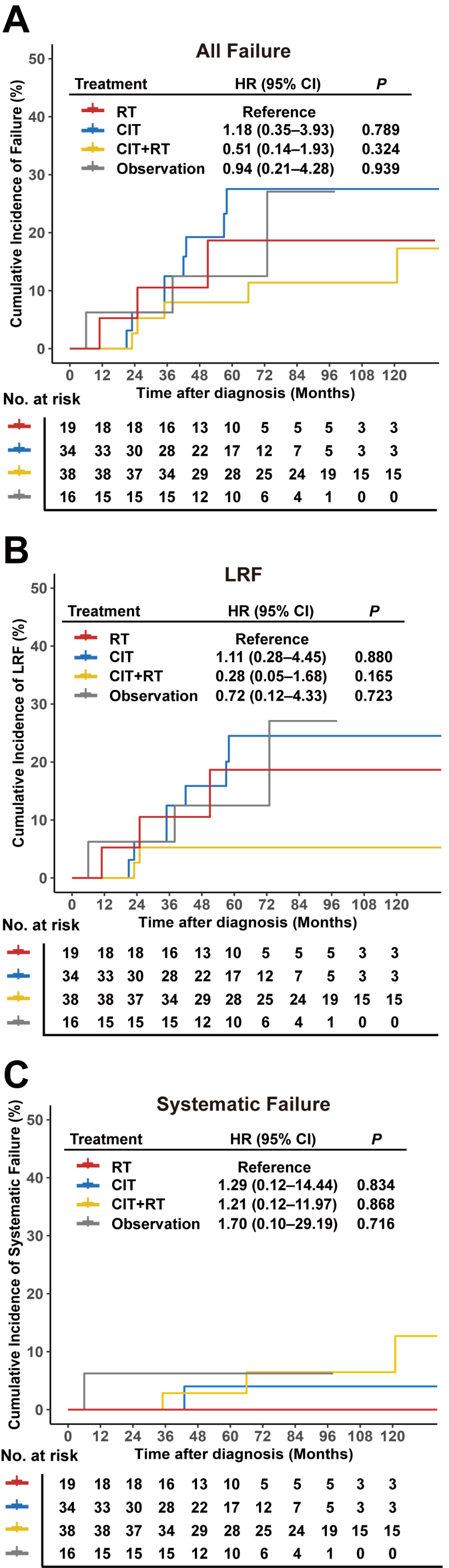
**

**Figure S2**


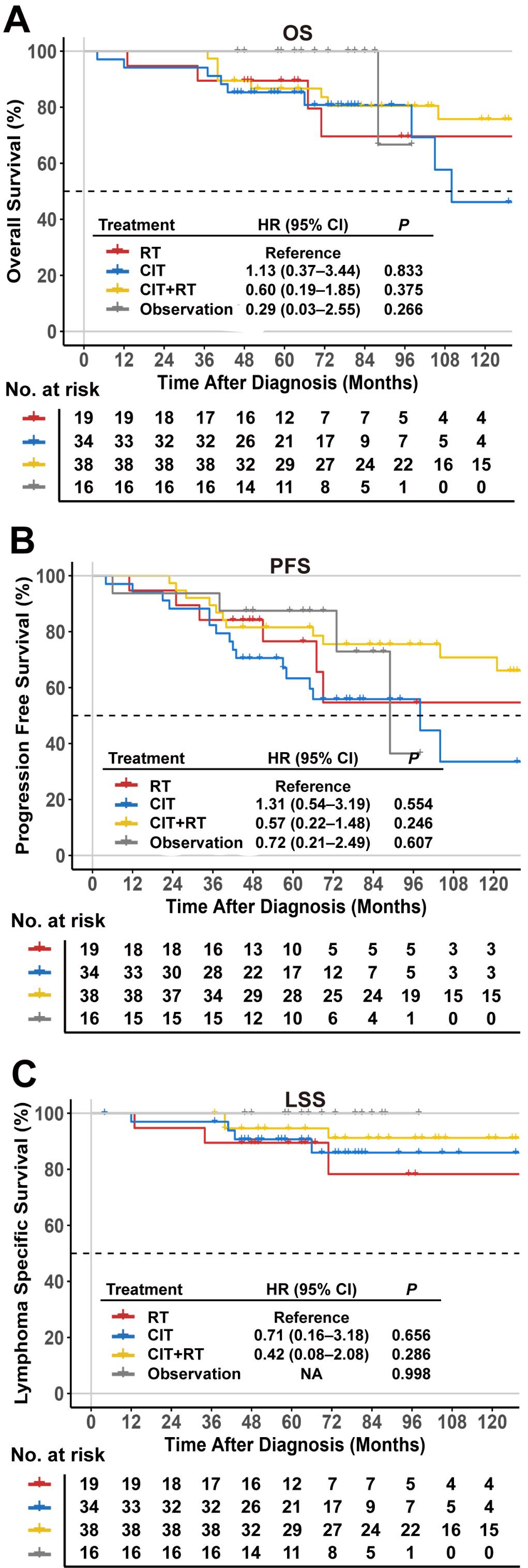


**Figure S3**

**
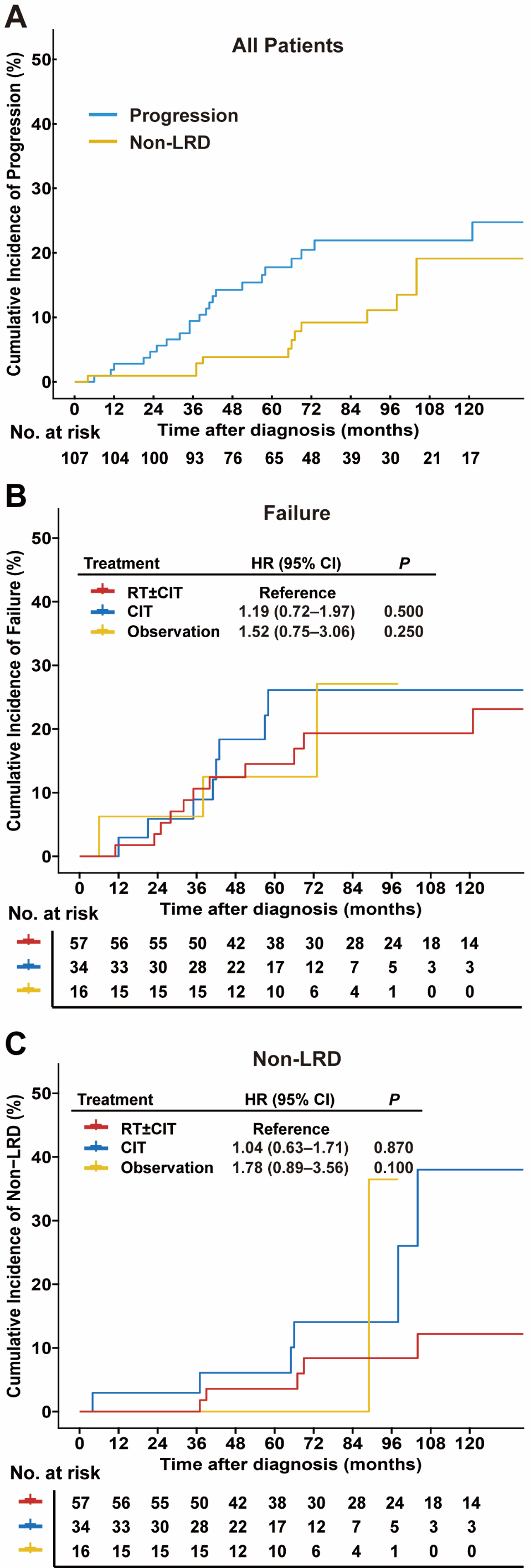
**

**Figure S4**

**
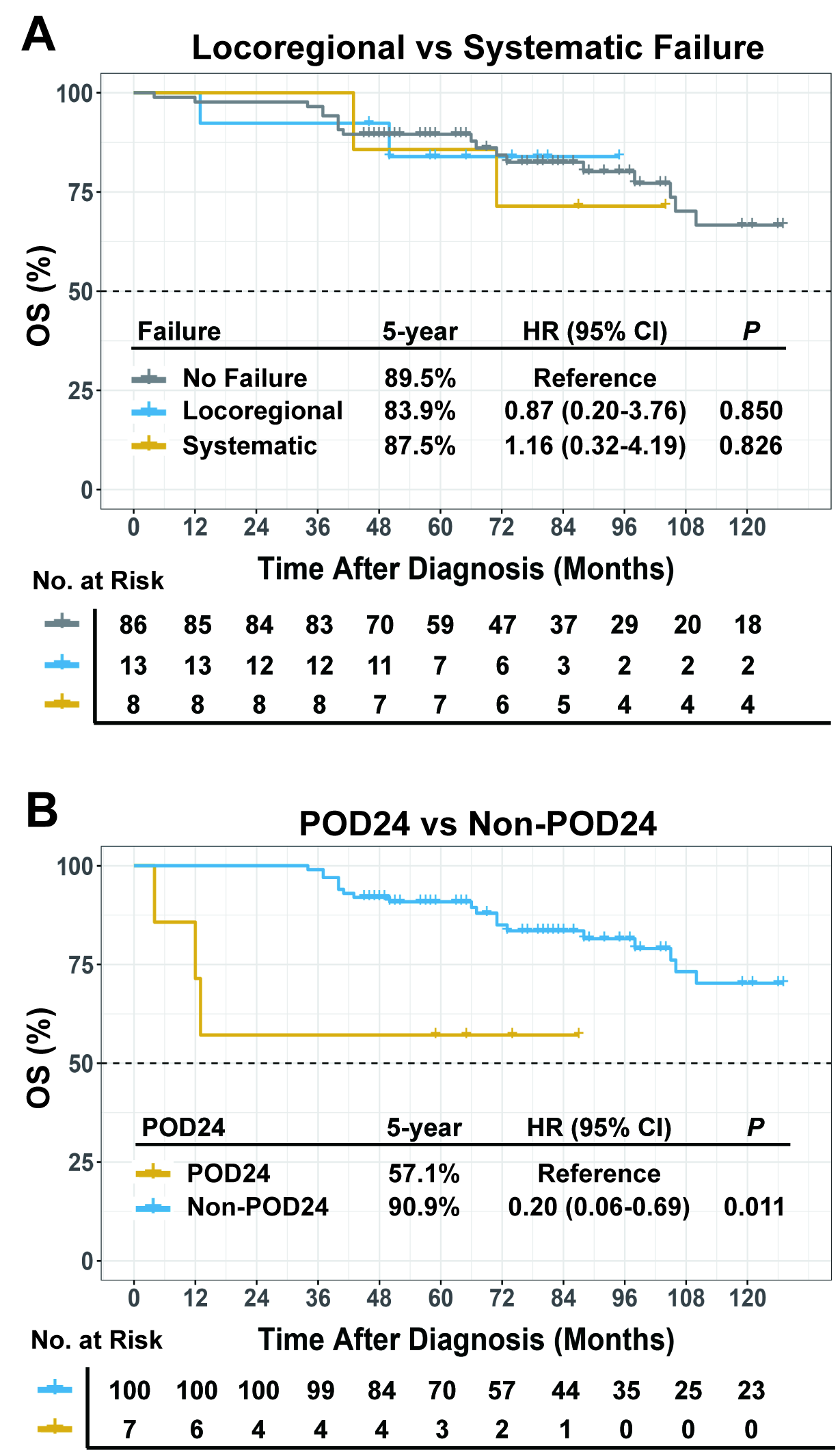
**

**Figure S5**

**
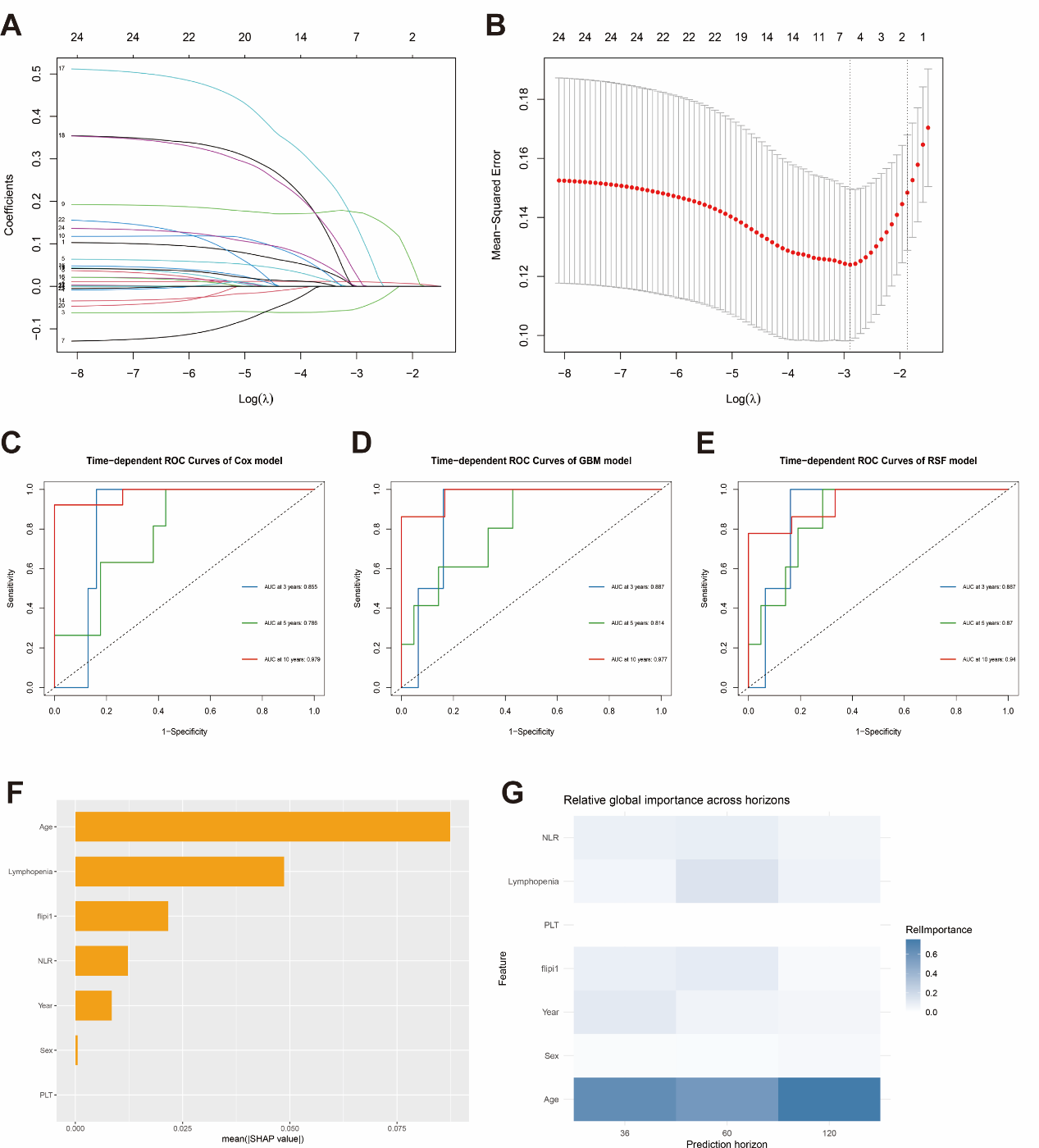
**
